# Prevalence of HCV and HIV infections in 2005-Earthquake-affected areas of Pakistan

**DOI:** 10.1186/1471-2334-8-147

**Published:** 2008-10-27

**Authors:** Saeed Khan, Mohammad A Rai, Adnan Khan, Amber Farooqui, Shahana U Kazmi, Syed H Ali

**Affiliations:** 1 Department of Biological and Biomedical Sciences, Aga Khan University Hospital, Karachi, Pakistan; 2 Immunology and Infectious Diseases Research Lab. (IIDRL) Department of Microbiology, Karachi University, Karachi, Pakistan

## Abstract

**Background:**

On October 8, 2005, an earthquake of magnitude 7.6 hit the Northern parts of Pakistan. In the post-earthquake scenario, overcrowding, improper sewage disposal, contamination of food and drinking water, hasty surgical procedures, and unscreened blood transfusions to earthquake victims most likely promotes the spread of infections already prevalent in the area.

**Objective:**

The objective of the study reported here was to determine the prevalence of Human Immunodeficiency and Hepatitis C viruses (respectively, HIV and HCV) in the earthquake-affected communities of Pakistan. The samples were analyzed 2 months and then again 11 months after the earthquake to estimate the burden of HIV and HCV in these areas, and to determine any rise in the prevalence of these viral infections as a result of the earthquake.

**Methods:**

Blood samples were initially collected during December, 2005 to March 2006, from 245 inhabitants of the earthquake-affected areas. These samples were screened for HCV and HIV, using immunochromatography and Enzyme-Linked Immuno-Sorbent Assay (ELISA).

**Results:**

Out of 245 samples tested, 8 (3.26%) were found positive for HCV, and 0 (0.0%) for HIV, indicating the existence of HCV infection in the earthquake-stricken areas. The same methods were used to analyze the samples collected in the second round of screening in the same area, in September, 2006 – 11 months after the earthquake. This time 290 blood samples were collected, out of which 16 (5.51%) samples were positive for HCV, and 0 for HIV.

**Conclusion:**

A slightly higher prevalence of HCV was recorded 11 months after the earthquake; this increase, however, was not statistically significant. None of the study participants was found HIV-infected.

## Background

Natural disasters generate mass casualty situations within a very short time [1-3]. Disasters such as earthquakes, tsunamis, and floods have an obvious immediate toll on human life and infra-structure. The gravity of such circumstances exacerbates due to the temporary paralysis of local emergency response and of healthcare services [4,5]. The issue of post-disaster management and care of the affected is equally important in addressing the prevention of infection and blood-borne diseases [2,6,7].

**Figure 1:**
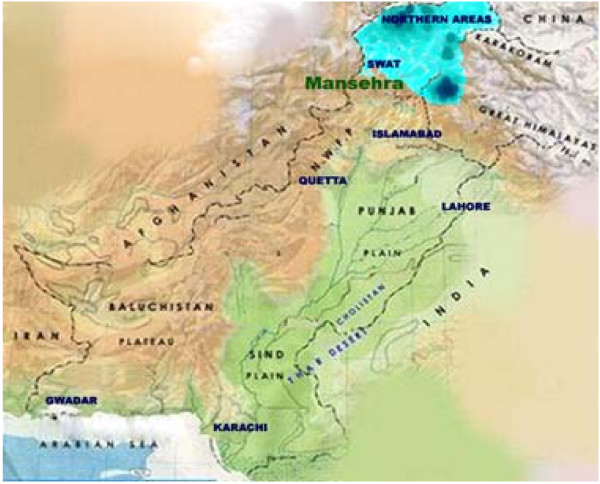
**Northern areas of Pakistan affected in the 2005 earthquake.** The affected areas are marked in blue. Intensity of blue corresponds to the recorded intensity of the earthquake in the area.

On October 8, 2005, at 08:50:38 am local time, a major earthquake measuring 7.6 on Richter scale hit the Northern areas of Pakistan. The epicenter of this earthquake was in Muzafarabad, about 95 kilometers Northeast of Pakistan's capital, Islamabad [8] (Fig. 1). As a result of this earthquake more than 100,000 lives were lost, and over three million people were left homeless at the mercy of freezing and harsh Himalayan winter [9].

Traumatic injuries contribute significantly to the mortalities incurred during an earthquake [10-12]. This is later followed by a significant increase in the transmission and spread of infectious diseases in the affected areas [13-15]. Conditions that facilitate spread of viral and other infections intensify in a post-disaster context. Displaced populations in camp settings are at high risk of infectious diseases owing to a large array of risk factors including inadequate shelter, overcrowding, inadequate quantity and quality of food, poor sanitation, poor personnel hygiene, economic and environmental degradation, compromised heathcare practices, and movement of people from areas of low to high endemicity [16]. Death rates of over 60 times the baseline have been recorded among refugees and displaced people, with over three-quarters of these deaths caused by communicable diseases [17].

Following the 2005 earthquake in Pakistan, local health care system in these Northern areas collapsed and help was summoned from all major cities of the country. International aid was also sought by the government, which was reciprocated significantly by the United Nations, the Red Crescent Society and other donor agencies. Not only did these relief organizations provide the necessary field and camp hospitals, but also offered the much-needed healthcare services to the affectees of the earthquake. Emergency health-providing facilities, clinics, operating rooms and laboratories were established in make-shift accommodations around the affected area [9].

The afore-mentioned risk factors were very much into play in the post-earthquake scenario in Northern Pakistan. Overcrowding of displaced population in camps settings, improper sewage disposal, contamination of food and scarcity of drinking water were commonly observed in these areas. Consequently, outbreaks of acute respiratory tract infections (ARTIs), scabies, diarrhea and other infections were recorded in the region [18].

The majority of the patients treated in the immediate aftermath of the earthquake had been admitted with fractures and injuries sustained during the earthquake [19]. In concordance with their constrained resources, the make-shift hospitals and camps offered a multitude of services, ranging from simple blood transfusions to complex orthopedic procedures. Therefore, services offered at these emergency medical facilities were at times unavoidably compromised. A great influx of patients along with scarcity of medical personnel and equipment, coupled with unscreened blood transfusion practices [20], were anticipated to promote the spread of blood-borne infections [20,21].

The objective of this study was to determine if the October earthquake led to an increase in the prevalence of HCV and HIV in the affected population. We analyzed random blood samples for these two infections from the affected population immediately after, and then 11 month following, the earthquake.

## Methods

### Study design and setting

This was a repeated cross sectional study in which the samples were initially collected during December, 2005 to March, 2006, starting six weeks after the earthquake. 245 study participants were recruited for this first stage of sampling. The second round of sample collection took place in September 2006, 11 months after the earthquake. The second round had a slightly greater number of participants, amounting to 290. The samples were collected from patients admitted to two camp hospitals in Mansehra, i.e., Italian Hospital in Mansehra and Saudi Field Hospital. Majority of these patients were presented with complaints of bone fractures and other injuries sustained during the earthquake. Some of the study subjects also reported to have undergone surgery and/or either received or donated blood during, or subsequently to, the earthquake aftermath.

### Study population

The study subjects came from all earthquake-affected Northern Pakistani regions, including the towns of Balakot, Mansehra, Batgram, Abbotabad, Garhi Habibullah and Muzaffarabad, among others.

The sample population was composed of patients and paramedical staff at different field hospitals set up in the region. These facilities had been established by Italy, Saudi Arabia and other national and international NGOs. In order to diversify the sample population, no restrictions were imposed while recruiting subjects. Consequently, the sample population included farmers, drivers, housewives, students, teachers, army personnel, health workers and others (Table 1).

**Table 1:** Distribution of subjects on the basis of Area of Residence, Gender, Age and Occupation

**Characteristic**	Bach A (n = 245)	Bach B (n = 290)	p-value
**Location**			
Mansehra	185 (75.5%)	250 (86.2%)	< 0.001
Abbotabad	03 (1.2%)	15 (5.1%)	
Balakot	14 (5.7%)	05 (1.7%)	
Batgram	07 (2.8%)	02 (0.6%)	
Other areas	36 (14.6%)	18 (6.2%)	
			
**Gender**			
Male	155 (63.2%)	136 (46.8%)	< 0.001
Female	90 (36.7%)	154 (53.1%)	
			
**Age**			
1–10 Years	18 (7.3%)	35 (12.0%)	0.166
11–25 Years	88 (35.9%)	105 (36.2%)	
26–50 Years	107 (43.6%)	124 (42.7%)	
Above 50 Years	32 (13.0%)	26 (8.9%)	
			
**Occupation**			
Army Personals	10 (4.0%)	5.0 (1.7%)	< 0.001
Drivers	17 (6.9%)	5.0 (1.7%)	
Farmers	38 (15.5)	20 (6.8%)	
House Wives	55 (22.4%)	98 (33.7%)	
Hospital Staff	30(12.2%)	50 (17.2%)	
Students and Teacher 59 (24.0%)	60 (20.6%)		
Others	36 (14.6%)	52 (17.9%)	

Frequencies for all values are given in parentheses. The p-values were calculated using Pearson chi square test.

### Specimen collection

Blood samples were collected aseptically in 5 ml red top vacutainers and left to clot. Sera specimens were separated after centrifugation, aliquoted into 2 ml Eppendorf tubes and stored at -20°C until the time for assay.

### Ethical issues

A written informed consent was obtained from the study participants prior to the collection of blood samples. The following information was given to each participant to ensure they were making an informed choice: a complete description of the aims of the study, infectious agents that were being screened, details of sample collection procedures, potential benefits and risks of their participation in the study, and assurance of confidentiality of any information given as well as of the test results. All donor information and test results were kept confidential.

### Detection of anti HIV-1 and HIV-2 antibodies

Detection of antibodies to HIV-1 and HIV-2 in the serum was done by rapid immunochromatography kit (Abbott, Japan) according to the manufacturer's guidelines. 50 μl of clear serum was applied to the sample pad with the help of a pipette and sterile tip. The results were noted after 15 minutes. Samples were tested twice for confirmation. Samples were retested on ELISA using SD HIV1/2 ELISA 3.0 (Standard Diagnostic, Korea). According to the information provided by manufacturers, for both the immunochromatography and SD HIV1/2 ELISA 3.0 tests, relative sensitivity and specificity were, respectively, 100% and 99.8%.

### Detection of anti HCV antibodies

Antibodies to HCV were detected using immunochromatography technique (Distinct, USA). According to the instructions of the manufacturer, 5 μl of clear serum was applied to the sample well followed by two drops of HCV buffer (provided in the kit), and the results were read within 10 minutes. The positive samples were tested twice for confirmation. Samples were retested on ELISA using SD HCV ELISA 3.0 (Standard Diagnostic, Korea). According to the information provided by manufacturers, for both the immunochromatography and SD HIV1/2 ELISA 3.0 tests, relative sensitivity and specificity were, respectively, 96–99% and 99%.

## Results

### Description of study population

We collected a total of 535 blood samples at two different time points from the 2005 earthquake victims in the Northern areas of Pakistan. The majority of the samples came from Mansehra (81%), Balakot, Abbotabad and other Northern areas of Pakistan (Fig. 1, Table 1). This distribution was applicable to both the first and second round of sample collection.

In the first round, 245 subjects were recruited. Of these, 155 (63.3%) were males and 90 (36.7%) were females, ratio, 1.7:1 (Table 1). Distribution of the group on the basis of age (Table 1) revealed that the majority 107 (43.6%) were in the age group 25–50 years. Adequate variability was represented in the samples by having people from different age groups. Distribution via occupation (Table 1) revealed a mixed picture; students and teachers numbered 59 (24%), housewives 55 (22.4%), with farmers, health care workers and others constituting the rest.

In the second round, 290 subjects were sampled. There were 136 males (46.9%) and a154 females (53.1%), ratio, 0.88:1 (Table 1). Distribution by age was similar to the first round (Table 1). Distribution by occupation (Table 1) showed the largest portion being composed of housewives (33.8%), with students and teachers, and healthcare workers constituting the rest.

When distribution of the above-mentioned parameters was examined in the two population samples, Pearson chi-square test returned p-values showing the two samples to be significantly different in terms of location, gender, and occupation, but not age (Table 1).

### Prevalence of HCV and HIV in the study population

In the first phase of study, in December 2005, out of the 245 samples, 8 (3.26%) were found positive by ELISA for HCV antibodies. Among the positive samples, male to female ratio was 3.2: 3.3. None of the samples were found positive for HIV by ELISA.

In the second round of sample collection, 11 months following the earthquake, another batch of 290 samples was analyzed. 13 of these subjects reported to have been injured during the earthquake and having received surgery, blood transfusion, and/or other forms of medical care. Out of the 290 samples analyzed, 16 (5.51%) were found positive by ELISA for HCV antibodies. This time, among the positive samples, male to female ratio was 1:2. Again, none of the samples were found positive for HIV using ELISA.

In our study, HCV infection was found in the earthquake-affected areas of Pakistan. The difference in the incidence of HCV in the two population samples was, however, found statistically nonsignificant by Pearson chi-square test (p-value > 0.1).

## Discussion and Conclusion

In the present study we have attempted to evaluate the effects of the earthquake of 2005, in the afflicted community residing in the Northern areas of Pakistan. We analyzed the prevalence, and any potential rise thereof following the disaster, of blood borne infections in the earthquake-afflicted communities HIV and HCV infections were used as indicators of blood-borne infections. We report here that no cases of HIV were found in the earthquake-affected areas immediately or 11 months following the disaster. We, however, recorded a 3.26% prevalence of HCV in the same population immediately following the disaster. Slightly higher (5.51%), albeit statistically non-significant, prevalence of HCV was recorded 11 months after the disaster.

Disasters are considered a major public health concern. They may cause an unexpected number of deaths, injuries, or illness in the affected community; may destroy local health infrastructures, or have adverse effects on the environment and the population, increasing the potential risk for communicable diseases and environmental hazards that will increase morbidity, premature death, and diminished quality of life in the future [14,21,22].

The October 8, 2005 Kashmir earthquake caused massive devastation and a considerable loss of human life. The healthcare services were hit particularly hard, with most of the hospice centers being either completely destroyed or seriously damaged. The affected population comprised mainly rural inhabitants, where health facilities were already lacking [23,24]. The quality of healthcare provided to the earthquake affectees was severely compromised. It has been reported that, the seven major hospitals in the area, only three had a functional blood transfusion service [25]. Quality assurance was very much lacking. Therefore, the risk of transmission for blood-borne infections in the after-care became an imminent threat.

Prevalence of Hepatitis C and HIV for the Northern parts of Pakistan is relatively unknown. No study has been conducted in the earth-quake affected region that gives us an idea of the status-quo regarding these two infections in particular, and blood-borne infections in general. Therefore, we undertook to investigate the baseline prevalence of these infections and then chart out any change that could be a potential consequence of the earthquake.

The reported Hepatitis C seroprevalence in Pakistan, determined from a sample over 47,000 individuals from the city of Islamabad, has been estimated 5.31% [26]. The only serological study in this direction, published in 2002, that involved the Northern areas of Pakistan revealed that 4% of healthy voluntary adults were positive for anti-HCV antibodies [27]. This issue is important because the risk factors for the spread of blood-borne infections are more common in the rural, under-developed Northern areas. The situation concerning HIV is also very similar. After the incidence of isolated outbreaks amongst the high-risk populations beginning in 2004, the need for demographic surveys has become all the more critical in Pakistan [28,29]. The only survey conducted amongst the people of the Northern areas reports the HIV sero-positivity 0.007% [27].

The purpose of this study was to establish a baseline first for the prevalence of Hepatitis C and HIV in the earthquake-affected population. Our analysis of the first batch of sampling revealed 3.26% of the population positive for HCV antibodies, and none for HIV. This incidence for HCV does not vary much from that reported earlier for other areas of Pakistan. The risk factors for the spread of HCV in this region; including unsafe injection practices, poverty, low literacy rate and poor health-seeking behavior have been charted out in the past [30]. The prevalence of HCV, therefore, was anticipated in these communities even before the advent of the earthquake.

We found no HIV prevalence in the second batch of samples collected 11 months after the earthquake, but the percentage of HCV positive samples was still found to be 5.51%. This rise in HCV prevalence was, however, not found statistically significant.

Pakistan has not as yet started experiencing the HIV epidemic [31]. Risk factors for HIV spread, particularly unawareness, low literacy rate, and practice of homosexuality, have been recorded in the Northern areas of Pakistan [32,33]. Moreover, the major bulk of HIV cases in Pakistan is comprised of deported migrant workers from the Gulf States, a significant proportion of whom are residents of Northern Pakistan [34]. A complete absence of HIV cases in our studied population was, therefore, surprising. The fact that our study was not able to detect any HIV infection may well be a result of the limited populace that was sampled. More expanded analysis is needed to truly chart the dynamics of HIV in these areas.

In contrast to HIV, a considerable prevalence of Hepatitis C was recorded in the earthquake-affected Northern areas. This observed HCV prevalence may be attributed to a varied mix of factors. The issue of unscreened blood transfusion ranks definitely as the most important contributory factor. Other factors may include the use of unsterilized equipment, lack of awareness and widespread illiteracy in Northern Pakistan. It may be speculated that compromised healthcare and other afore-mentioned factors related to earthquake will aggravate the transmission and prevalence scenario in these areas. The slight increase observed in the two batches studied may be attributed to the variation in the population samples studied in the two instances. The two sample populations indeed were significantly different according to most of the measured parameters (Table 1).

At this time, proper guidelines and procedures, implemented urgently, could play a significant role in reducing the spread of blood-borne infections. Mandatory screening of blood and blood products before transfusion, proper sterilization of surgical and dental instruments, and appropriate disposal of infected materials and disposable syringes are among the issues that need to be emphasized [21,35,36].

## Competing interests

The authors declare that they have no competing interests.

## Authors' contributions

SA carried out all the experimental work and helped in writing the manuscript. MAR helped with data interpretation and manuscript writing. AK and AF assisted SA in the collection of blood samples and in the administration of questionnaire survey. SUK supervised the work in Mansehra and arranged for partial funding. SHA supervised the work in Karachi, arranged for partial funding and helped in the writing of manuscript. All authors have read and approved the final manuscript.

## Pre-publication history

The pre-publication history for this paper can be accessed here:


